# The derived allele of a novel intergenic variant at chromosome 11 associates with lower body mass index and a favorable metabolic phenotype in Greenlanders

**DOI:** 10.1371/journal.pgen.1008544

**Published:** 2020-01-24

**Authors:** Mette K. Andersen, Emil Jørsboe, Line Skotte, Kristian Hanghøj, Camilla H. Sandholt, Ida Moltke, Niels Grarup, Timo Kern, Yuvaraj Mahendran, Bolette Søborg, Peter Bjerregaard, Christina V. L. Larsen, Inger K. Dahl-Petersen, Hemant K. Tiwari, Bjarke Feenstra, Anders Koch, Howard W. Wiener, Scarlett E. Hopkins, Oluf Pedersen, Mads Melbye, Bert B. Boyer, Marit E. Jørgensen, Anders Albrechtsen, Torben Hansen

**Affiliations:** 1 Novo Nordisk Foundation Center for Basic Metabolic Research, Faculty of Health and Medical Sciences, University of Copenhagen, Copenhagen, Denmark; 2 The Bioinformatics Centre, Department of Biology, University of Copenhagen, Copenhagen, Denmark; 3 Department of Epidemiology Research, Statens Serum Institut, Copenhagen, Denmark; 4 PEPperPRINT GmbH, Heidelberg, Germany; 5 National Institute of Public Health, University of Southern Denmark, Copenhagen, Denmark; 6 Greenland Centre for Health Research, University of Greenland, Nuuk, Greenland; 7 Department of Biostatistics, School of Public Health, University of Alabama at Birmingham, Birmingham, Alabama, United States of America; 8 Department of Infectious Diseases, Rigshospitalet University Hospital, Copenhagen, Denmark; 9 Department of Epidemiology, School of Public Health, University of Alabama at Birmingham, Birmingham, Alabama, United States of America; 10 Department of Obstetrics and Gynecology, Center for Developmental Health, Knight Cardiovascular Institute, Oregon Health & Science University, Portland, Oregon, United States of America; 11 Center for Alaska Native Health Research, University of Alaska Fairbanks, Fairbanks, Alaska, United States of America; 12 Department of Clinical Medicine, Faculty of Health and Medical Sciences, University of Copenhagen, Copenhagen, Denmark; 13 Department of Medicine, Stanford University School of Medicine, Stanford, California, United States of America; 14 Steno Diabetes Center Copenhagen, Gentofte, Denmark; 15 Faculty of Health Sciences, University of Southern Denmark, Odense, Denmark; NIDDK Phoenix Branch, UNITED STATES

## Abstract

The genetic architecture of the small and isolated Greenlandic population is advantageous for identification of novel genetic variants associated with cardio-metabolic traits. We aimed to identify genetic loci associated with body mass index (BMI), to expand the knowledge of the genetic and biological mechanisms underlying obesity. Stage 1 BMI-association analyses were performed in 4,626 Greenlanders. Stage 2 replication and meta-analysis were performed in additional cohorts comprising 1,058 Yup’ik Alaska Native people, and 1,529 Greenlanders. Obesity-related traits were assessed in the stage 1 study population. We identified a common variant on chromosome 11, rs4936356, where the derived G-allele had a frequency of 24% in the stage 1 study population. The derived allele was genome-wide significantly associated with lower BMI (beta (SE), -0.14 SD (0.03), p = 3.2x10^-8^), corresponding to 0.64 kg/m^2^ lower BMI per G allele in the stage 1 study population. We observed a similar effect in the Yup’ik cohort (-0.09 SD, p = 0.038), and a non-significant effect in the same direction in the independent Greenlandic stage 2 cohort (-0.03 SD, p = 0.514). The association remained genome-wide significant in meta-analysis of the Arctic cohorts (-0.10 SD (0.02), p = 4.7x10^-8^). Moreover, the variant was associated with a leaner body type (weight, -1.68 (0.37) kg; waist circumference, -1.52 (0.33) cm; hip circumference, -0.85 (0.24) cm; lean mass, -0.84 (0.19) kg; fat mass and percent, -1.66 (0.33) kg and -1.39 (0.27) %; visceral adipose tissue, -0.30 (0.07) cm; subcutaneous adipose tissue, -0.16 (0.05) cm, all p<0.0002), lower insulin resistance (HOMA-IR, -0.12 (0.04), p = 0.00021), and favorable lipid levels (triglyceride, -0.05 (0.02) mmol/l, p = 0.025; HDL-cholesterol, 0.04 (0.01) mmol/l, p = 0.0015). In conclusion, we identified a novel variant, where the derived G-allele possibly associated with lower BMI in Arctic populations, and as a consequence also leaner body type, lower insulin resistance, and a favorable lipid profile.

## Author summary

The risk of developing obesity is strongly affected by lifestyle, particularly diet and level of physical activity, but also by genetic predisposition. Knowledge about the genes predisposing to obesity can inform about biological processes underlying this condition, and possibly identify targets for obesity treatment. In the present study, we take advantage of the genetic architecture of the Greenlandic population to identify genetic variants associated with alterations in body-mass index, as a measure of obesity. By examining more than 100,000 genetic variants in 4,626 Greenlanders we identify a specific variant, rs4936356, where the derived G-allele was associated with lower body-mass index, lower insulin resistance, and favorable lipid levels. We verified the association with body-mass index in a combined analysis including two additional Arctic cohorts. These results contribute to the understanding of the genetic predisposition to obesity, however, further studies are required to replicate these findings and to identify the gene through which the rs4936356 variant is affecting body-mass index.

## Introduction

Obesity is an increasing health problem worldwide. The condition is caused by a combination of environmental risk factors and genetic predisposition. Identification of genetic variants associated with obesity could, therefore, lead to improved understanding of mechanisms underlying this condition, and thereby identification of possible targets for prevention and treatment. To date, more than 900, mostly common, gene variants associated with obesity have been identified in genome-wide association studies (GWAS), assessing body-mass index (BMI) as a surrogate measure of obesity [1,2]. Despite the high number of loci, the identified variants explain only ~6% of the BMI variance [1,2], indicating that there are additional variants to be found. These unidentified variants are likely either of too low frequency or have too small effect sizes in the studied populations to be identified with the current sample sizes and analysis strategies. The primary strategy, until now, has been to perform the association studies in large outbred populations like Europeans, North Americans, and Asians. An alternative strategy, which may facilitate discovery of additional variants, is to perform the association studies in isolated populations, like the Greenlandic. Compared to large outbred populations, isolated populations show extended patterns of linkage disequilibrium (LD), and a higher probability for the presence of disease-associated variants with high frequency due to genetic drift and selection [3,4]. These properties are advantageous for genetic-association studies, which have recently been demonstrated in various isolated populations by the discovery of novel variants associated with cardio-metabolic traits [5–13], and of particular interest coding variants in *CREBRF* and *ADCY3* have been associated with obesity in Samoans and Greenlanders, respectively [14,15].

The Greenlandic population has evolved under conditions characterized by interchanging periods of feast and famine, where fat accumulation and post-prandial insulin resistance [5,16] may have been advantageous in order to maximize the utility of the available food resources. However, with the rapid lifestyle transition, and increasing food availability over the last 60 years, the obesity prevalence in Greenland has increased dramatically. In 2018, 24% of Greenlandic men and 32% of women were obese [17], similar to numbers reported for European and North American populations [18]. Improving the understanding of the mechanisms leading to obesity in the Greenlandic population is, thus, of major importance.

In the present large-scale association study, we aim to identify novel genetic loci associated with obesity and related metabolic phenotypes in Greenlanders, and thereby possibly gain further insight into the genetics underlying this condition.

## Results

### Stage 1–BMI-association analyses in Greenlanders

**Fig 1:**
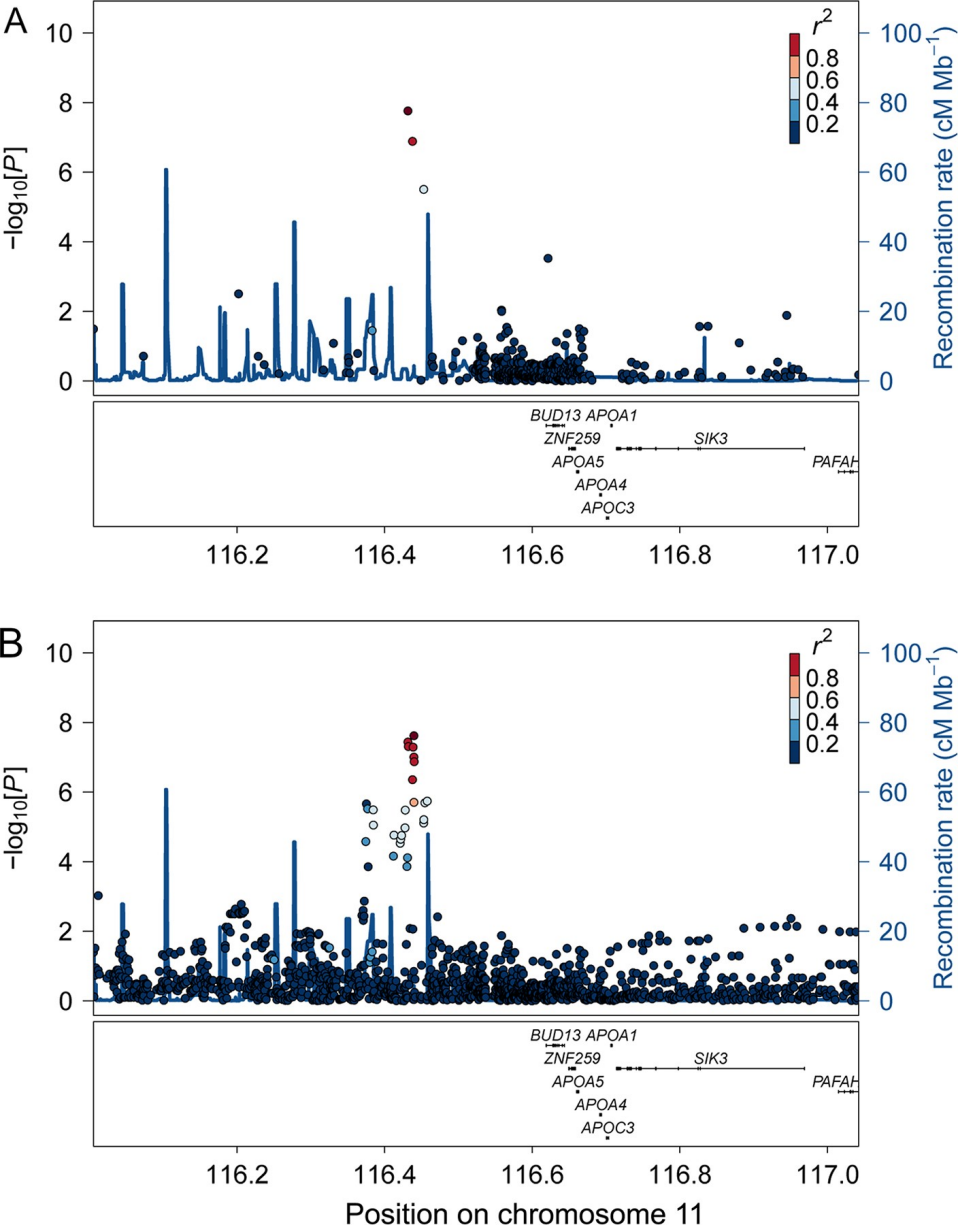
Regional BMI-association results. The plots are based on Metabochip data (A) or imputed data (B). The dark red dot in each plot indicates the lead SNP in the region (A, rs4936356; B, rs7928307), the rest of the SNPs are colored according to the extent of correlation (*r*^2^) with the respective lead SNP.

**Fig 2:**
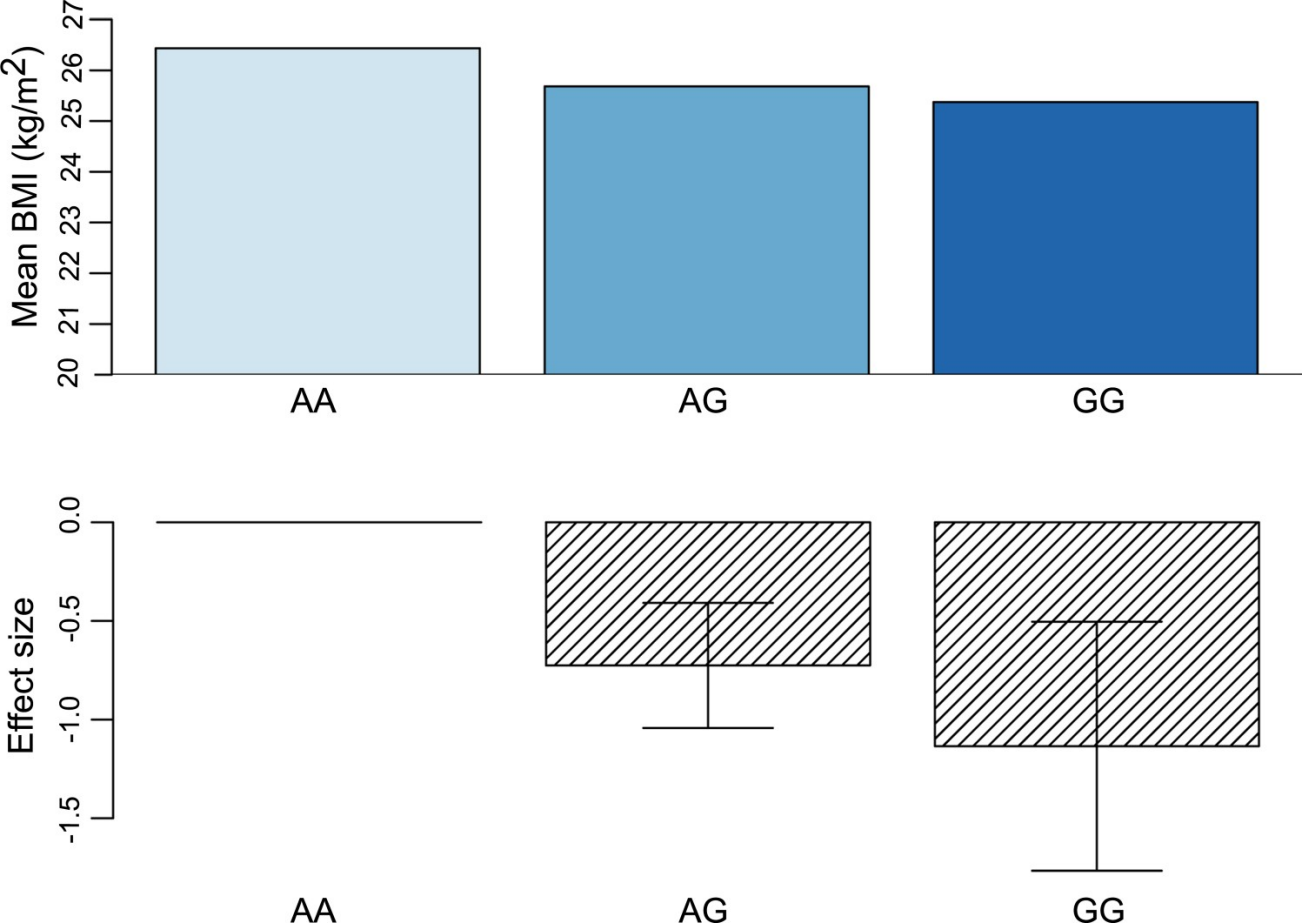
Additive effect of rs4936356 on BMI. The mean BMI and effect estimates ±s.e.m. from the additive genetic model are shown according to rs4936356 genotype.

In BMI-association analyses applying an additive genetic model on 115,182 variants from the Metabochip, one locus on chromosome 11 reached genome-wide significance in the stage 1 analysis (Figs 1A and S1). Extending the analysis by applying a recessive genetic model did not identify additional loci associated with BMI (S2 Fig). The most significant variant in the locus on chromosome 11 from the additive association analysis was the intergenic rs4936356 variant, where the derived G-allele was associated with lower BMI (beta SD (se), -0.14 SD (0.03), p = 3.2x10^-8^), corresponding to 0.64 (SE, 0.13) kg/m^2^ lower BMI per G-allele (Table 1 and Fig 2). The estimated effect size according to rs4936356 genotype suggested that the effect of the variant on BMI was additive (Fig 2).

**Table 1:** Association of rs4936356 with body-mass index in Arctic cohorts.

	N	β (SD)	SE	p-value
**Stage 1**				
Greenlanders	4,626	-0.14	0.03	3.2x10^-8^
**Stage 2**				
Yup’ik Alaska Native people	1,058	-0.09	0.04	0.038
Greenlanders	1,529	-0.03	0.04	0.514

Results are shown for an additive genetic model, where the effect size and p-values were obtained based on quantile transformed values of the trait. SD, standard deviation; SE, standard error.

To fine map the region on chromosome 11 harboring rs4936356, we assessed imputed data. The imputation-based analyses revealed additional non-coding SNPs in the locus (Fig 1B). However, only one of the imputed SNPs (rs7928307) had a slightly lower p-value than rs4936356. High LD between rs7928307 and rs4936356 (R^2^ = 0.86 and D’ = 1.0), and association analyses conditional on rs4936356 indicated that the variants represent the same association signal (S3 Fig). Hence, we based the follow-up analyses on the genotyped (rs4936356) rather than an imputed variant. The derived G-allele of rs4936356 had a frequency of 24% (95% CI, 23–25%) in the Greenlandic study population. Notably, the Greenlandic population is an admixture of Inuit and Europeans and the derived G-allele of rs4936356 was estimated to be more frequent in the Inuit ancestry component of the population, with a frequency of 28% (27–29%), compared to the European ancestry component with a frequency of 15% (12–18%). For comparison, in the 1000 genomes project the observed frequency of the rs4936356 G-allele in the British (GBR) and Europeans from Utah (CEU) populations was 9.2% and 6.6%, respectively. Analyses estimating the effect in each ancestry component of Greenlanders separately, applying the Asamap method [19], suggested that the observed effect on BMI was mainly driven by the Inuit compared to the European component (beta SD (SE), -0.16 SD (0.04), p = 2.9x10^-6^ vs. -0.03 SD (0.13), p = 0.77); however, the effect did not differ significantly between the two population components (p = 0.36).

### Stage 2–replication and meta-analysis of BMI association

To verify our findings, we assessed the rs4936356-BMI association in 1,058 Alaska Native Yup’ik people, and in an independent cohort of 1,529 Greenlanders. The frequency of the rs4936356 G-allele, was similar in stage 1 and stage 2 Greenlandic cohorts (24% and 26%, respectively), whereas we observed a lower frequency among the Yup’ik participants of 14%.

**Fig 3:**
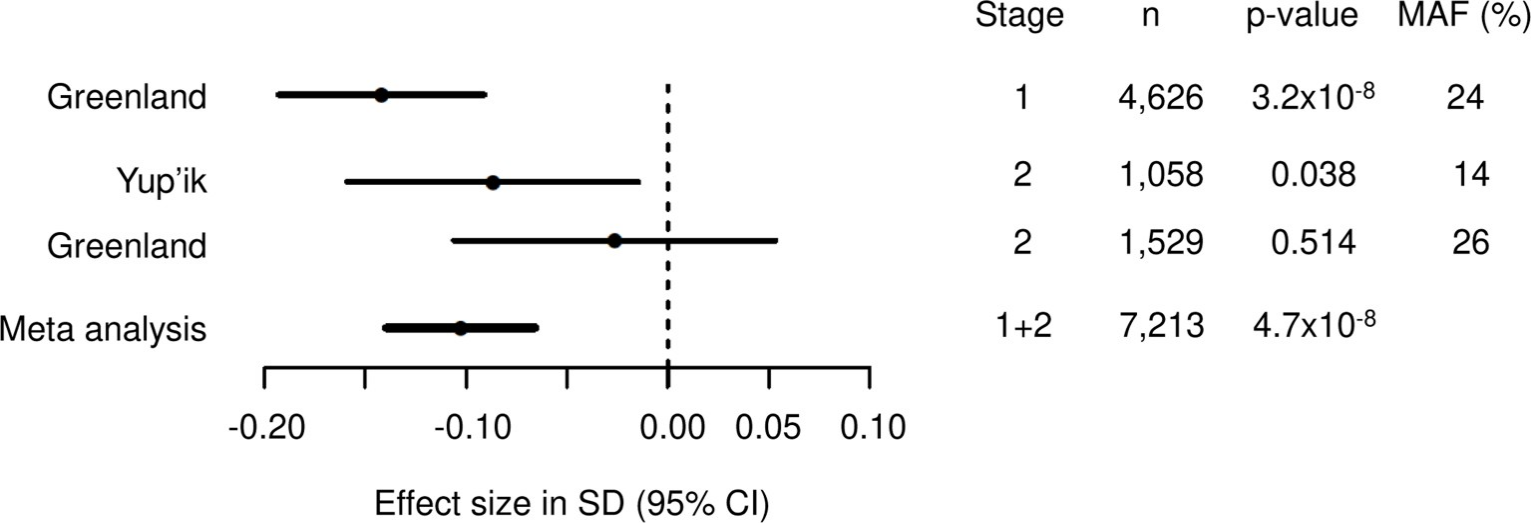
Forest plot of association between rs4936356 and BMI. Fixed-effect meta-analysis of 7,213 individuals from three different Arctic study populations. Heterogeneity between the populations was assessed with Cochrane’s Q (p = 0.05).

When attempting to replicate the BMI association in the stage 2 study populations, we observed a similar effect in the Yup’ik participants (beta SD (se), -0.09 SD (0.04), p = 0.038), but a smaller, if any, effect in the Greenlanders (-0.03 SD (0.04), p = 0.514) (Table 1). Combining stage 1 and stage 2 study populations in a meta-analysis supported the genome-wide significant association of rs4936356 with BMI (-0.10 SD (0.02), p = 4.7x10^-8^) (Fig 3).

Moreover, to explore whether the observed association could be generalized to Europeans, we assessed the effect of rs4936356 on BMI in European GWAS summary statistics [2]. In this data set, comprising around 450,000 UK Biobank participants and around 250,000 Europeans from the GIANT consortium, the rs4936356 G-allele had a frequency of 7% and was nominally associated with lower BMI (-0.009 SD (0.003), p = 0.0056).

### Association analyses of obesity-related traits in Greenlanders

In addition to the association with lower BMI, the rs4936356 G-allele was also associated with other measures of a leaner body type in the stage 1 Greenlandic study population. Significant associations included lower weight (beta (se), -1.68 (0.37) kg, p = 6.7x10^-7^), waist (-1.52 (0.33) cm, p = 1.4x10^-6^), hip (-0.85 (0.24) cm, p = 2.7x10^-5^), lean mass (-0.84 (0.19) kg, p = 1.9x10^-6^), fat mass and percent (-1.66 (0.33) kg, p = 3.2x10^-8^ and -1.39 (0.27) %, p = 1.1x10^-7^), visceral adipose tissue (-0.30 (0.07) cm, p = 1.6x10^-5^), and subcutaneous adipose tissue (-0.16 (0.05) cm, p = 0.0002). In line with the leaner body type, the variant was also nominally associated with a better metabolic profile with lower insulin resistance (HOMA-IR, -0.12 (0.04), p = 0.0002), and favorable lipid levels (triglycerides, -0.05 (0.02) mmol/l, p = 0.025; HDL-cholesterol, 0.04 (0.01) mmol/l, p = 0.0015). However, these associations all seemed to be driven by BMI, as none of them remained significant after adjusting the association analyses for BMI (Table 2).

**Table 2:** Association of rs4936356 with quantitative metabolic traits in Greenlanders.

Trait	N	β (SD)	SE	β (trait unit)	p-value	p-value_BMIadj
**Body composition**						
BMI (kg/m^2^)	4626	-0.14	0.03	-0.64	3.2x10^-8^	NA
Height (cm)	4648	-0.01	0.02	-0.03	0.793	0.979
Weight (kg)	4631	-0.13	0.03	-1.68	6.7x10^-7^	0.233
Waist (cm)	4594	-0.12	0.03	-1.52	1.4x10^-6^	0.128
Hip (cm)	4592	-0.11	0.03	-0.85	2.7x10^-5^	0.601
Lean mass (kg)	2702	-0.14	0.03	-0.84	1.9x10^-6^	0.143
Fat mass (kg)	2702	-0.18	0.03	-1.66	3.2x10^-8^	0.019
Fat (%)	2713	-0.17	0.03	-1.39	1.1x10^-7^	0.105
VAT (cm)	2693	-0.14	0.03	-0.30	1.6x10^-5^	0.169
SAT (cm)	2683	-0.12	0.03	-0.16	0.0002	0.816
**Glucose homeostasis**						
Fp glucose (mmol/l)	3693	-0.05	0.03	-0.04	0.059	0.798
2h-p glucose (mmol/l)	3437	-0.01	0.03	-0.01	0.770	0.419
HbA1c (%)	4624	-0.01	0.02	-2.6x10^-4^	0.568	0.411
**Insulin sensitivity**						
Fs insulin (pmol/l)	3691	-0.11	0.03	-2.16	0.0002	0.368
2h-s insulin (pmol/l)	3437	-0.03	0.03	-0.53	0.310	0.743
HOMA-IR (mmol/lxpmol/l)	3684	-0.10	0.03	-0.12	0.0002	0.380
**Lipids**						
Fs triglyceride (mmol/l)	4124	-0.06	0.03	-0.05	0.025	0.504
Fs HDL-cholesterol (mmol/l)	4652	0.08	0.03	0.04	0.0015	0.229
Fs cholesterol (mmol/l)	4517	0.03	0.03	0.03	0.279	0.06

Results are shown for an additive genetic model. β (SD) is the effect size estimated using quantile transformed values of the trait, and β (trait unit) is the effect size estimated using untransformed values. The p-values were obtained from the quantile transformed value based analyses, with (p-value) or without BMI (p-value_BMIadj) included as a covariate. F, fasting; HOMA-IR, homeostasis model assessment of insulin resistance; NA, not applicable; p, plasma; SAT, subcutaneous adipose tissue; s, serum; SD, standard deviation; SE, standard error; VAT, visceral adipose tissue.

### Functional assessment of rs4936356

#### Causal variant

Based on assessment of the possible functional impact of rs4936356, via RegulomeDB [20] and HaploReg V4.1 [21], we were unable to determine whether rs4936356 could be the causal variant in the locus, as no major effects on regulatory elements in the region were apparent. However, in our data, we were unable to identify a better candidate for the causal variant in the locus.

#### Causal transcript

**Fig 4:**
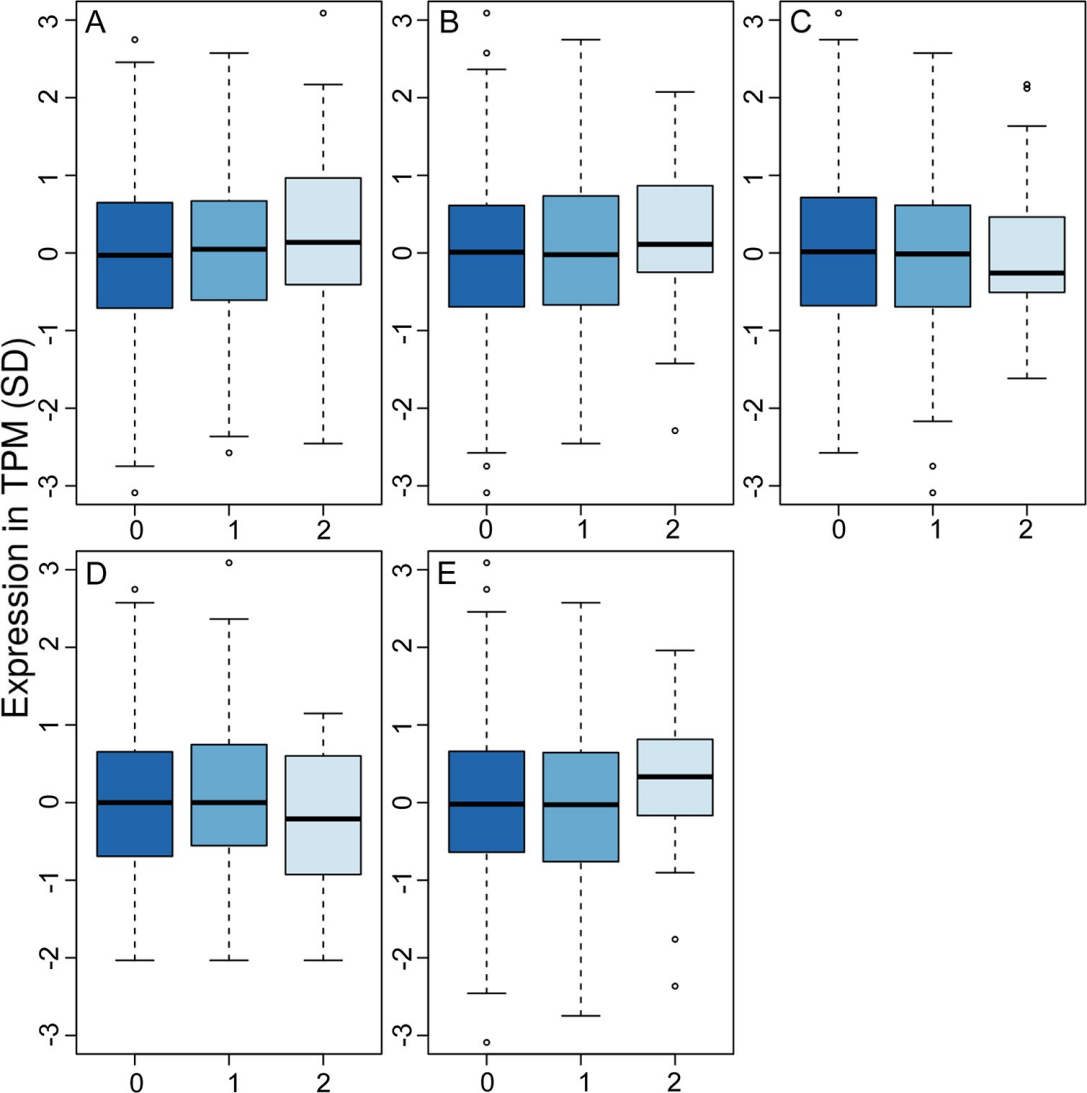
RNA expression of genes near rs4936356. Expression of A) *CADM1*, B) *BUD13*, C) *ZNF259*, D) *SIK3*, and E) *APOA1* analyzed in blood samples from 499 Greenlanders. The expression is displayed as transcripts per million (TPM (SD)) according to the number of rs4936356 G-alleles. Possible differences in expression according to rs4936356 genotype was assessed by a linear mixed model adjusting for age and sex (p>0.05 for all genes).

In an attempt to identify the causal gene in the locus, we assessed RNA expression data in leukocytes from 499 Greenlanders from the stage 1 study population. We looked at the expression of genes near rs4936356, including *BUD13*, *ZNF259*, *APOA5*, *APOA4*, *APOC3*, *APOA1*, and *SIK3* upstream, and *CADM1* downstream, of the variant. However, expression of *APOA4*, *APOA5*, and APOC3 was not observed in blood, and none of the remaining genes showed altered expression according to rs4936356 genotype (Fig 4). In line with this, rs4936356 did not affect the expression of any of the mentioned genes across 48 tissues assessed via the GTEx portal (https://www.gtexportal.org/). Of note, based on the Metabochip data and imputed variants in the region, LD between rs4936356 and SNPs in or near any of these genes seemed to be low (r^2^<0.2) in the Greenlandic study population.

## Discussion

In Greenlanders, we identified an intergenic variant in a novel locus, rs4936356 on chromosome 11, where the derived G-allele was significantly associated with lower BMI, and as a consequence of the lower BMI also a leaner body composition, and a more favorable metabolic profile in terms of levels of insulin resistance, and circulating lipids. The effect of rs4936356 on BMI was additive, and applying a recessive genetic model did not reveal additional BMI-associated loci. The novel BMI-association signal is independent of variants previously reported to be associated with obesity in Europeans [1,2]. The signal was marginally replicated in a cohort of 1,058 Yup’ik Alaska Native people, and we observed a non-significant effect on BMI, in the same direction, in additional 1,529 Greenlanders. The BMI association remained significant when combining data from all three Arctic cohorts in a meta-analysis, however, the effect sizes where smaller in the replication cohorts, which might be explained by winner’s curse causing an overestimation of the effect size in the discovery cohort. In Europeans, we observed borderline replication of the association with BMI, thus indicating that the association can possibly be generalized to other populations. The more modest association observed in Europeans could be due to the lower effect allele frequency in this population, compared to Greenlanders, particularly those of Inuit ancestry, or it could be due to population-dependent differences in LD between rs4936356 and the causal variant in the region. The rs4936356 variant adds to the picture of a markedly different genetic architecture of complex traits in isolated populations compared to Europeans, as rs4936356 is common in the investigated isolated Arctic populations and have a relative large effect on BMI, compared to common variants associated with BMI in Europeans [2,22]. This difference in genetic architecture of metabolic traits is also supported by recent studies identifying common variants associated with BMI with a large effect sizes in Samoans and Greenlanders, respectively [14,15].

Despite querying RNA expression data both from blood samples from Greenlanders and from multiple tissues from Europeans [23,24], we failed to identify a possible causal transcript in the locus. This could be due to the fact that rs4936356 is not the causal variant, or due to lack of analyses of relevant tissues, like the brain or adipose tissue, in a sufficient number of samples. The locus contains a number of interesting candidate genes, including the apolipoprotein genes and *SIK3*, encoding the salt-inducible kinase 3 (SIK3). Variants in the apolipoprotein genes in the locus, namely *APOA1*, *APOA4*, *APOA5*, and *APOC3*, have previously been linked to circulating levels of different lipids [25–27], but not BMI [2]. The protein encoded by *SIK3* belongs to the 5’-AMP-activated protein kinase (AMPK)-related kinase family [28], a protein family related to AMPK, which is a master regulator of metabolism [29]. Functional studies and model organisms strongly support *SIK3* as a biological candidate gene in the region. In *C*. *elegans*, mutation of the SIK3 orthologue, kin-29, has been linked to small body size [30], and in *Drosophila*, SIK3 has been linked to regulation of lipid metabolism [31], regulation of energy balance [32], and maintenance of glucose tolerance [33]. *Sik3*^*-/-*^ mice display lipodystrophy, hypolipidemia, hypoglycemia, and hyper-insulin sensitivity [34,35]. Moreover, the lack of Sik3 in mice was linked to reduced energy storage, and resistance to weight gain from a high-fat diet [34]. The described phenotypes of knock-out mice, and other model animals, match our observations of reduced body size, lower levels of triglycerides, and higher insulin sensitivity in carriers of the rs4936356 G-allele.

We have no direct evidence for a link between rs4936356 and a causal variant affecting the expression or function of SIK3. The genomic distance between rs4936356 and *SIK3* is 412-667Kb, which is longer than the estimated extent of LD in general human populations [36]. Interestingly, among Greenlanders, LD across much larger distances has been described [6,37,38], hence, in this population, it is possible that *SIK3* is the causal gene despite the distance to the identified marker.

Enhanced utilization of fat and glucose, instead of storage, as well as hyper-insulin-sensitivity, may contribute to the mechanisms underlying the observed phenotype in our study. It is possible that enhanced ability to utilize fat would have been evolutionarily favorable in the Greenlandic population that historically has adapted to a lifestyle with limited food supplies, extended periods of fasting, and a diet rich in omega-3 fatty acids [39]. Previous studies have shown that the Greenlandic population history has shaped the genetic landscape [37,40,41], and it is therefore also likely that it may have had an effect on the prevalence of the causal variant in the identified locus.

In conclusion, we identified a novel locus on chromosome 11, where the derived allele possibly was associated with lower BMI, and therefore also a leaner body type, lower insulin resistance, and a favorable lipid profile. Even though we failed to identify the causal variant and transcript in the region, our findings may have clinical implications as the locus could be a therapeutic target for improved metabolic health. Additional studies focusing on replication as well as fine mapping of the region to identify the causal variant, and studies assessing expression profiling across tissues to identify the causal transcript, are warranted.

## Materials and methods

### Ethics statement

All participants gave written informed consent. The stage 1 study was approved by the Commission for Scientific Research in Greenland (project 2011–13, ref. no. 2011–056978; and project 2013–13, ref.no. 2013–090702), and the study was conducted in accordance with the ethical standards of the Declaration of Helsinki, second revision. The stage 2 Yup’ik study protocols were approved by the Institutional Review Boards of the University of Alaska Fairbanks, and the National and Alaska Area Indian Health Service Institutional Review Boards, as well as the Yukon-Kuskokwim Health Corporation Human Studies Committee [42]. The stage 2 Greenlandic study was approved by the Commission for Scientific Research in Greenland (approval No. 2013–17), and the Danish Data Protection Agency.

### Stage 1 study population

The study population for the stage 1 association analysis comprised Greenlanders from three cohorts, Inuit health in transition (IHIT; n = 3,115), B99 (n = 1,401), and BBH (n = 547). During 1999–2001 and 2005–2010, respectively, the B99 and IHIT cohorts were collected as part of a general population health survey of the Greenlandic population, as described in [43,44]. BBH comprises Greenlanders living in Denmark, and was collected during 1998–1999 [43]. There was an overlap of 295 individuals examined both in IHIT and B99, these individuals were assigned to B99.

### Stage 2 study populations

The stage 2 study population comprised two cohorts of 1,480 Yup’ik Alaska Native individuals and 1630 Greenlanders, respectively. The Yup’ik individuals were 14 years or older, and were recruited by the Center for Alaska Native Health Research from 11 Southwest Alaska communities. The Greenlanders were 16 years or older, and participant samples were collected as a population-based sample from seven towns [45]. There was an overlap of 41 individuals between stage 1 and stage 2 Greenlandic cohorts, these individuals were assigned to the stage 1 cohort.

### Measurements and assays

For all included individuals height and weight were measured, and BMI calculated as weight in kilograms divided by height in meters squared. Moreover, additional phenotypes were collected for the Greenlanders in the stage 1 study sample. We measured the waist circumference midway between the rib cage and the iliac crest, and hip circumference at its maximum while participants were standing upright. All IHIT participants above 18 years, and B99 participants above 35 years, underwent an oral glucose tolerance test, where blood samples were drawn after an overnight fast of at least 8 hours, and 2 hours after receiving 75 g glucose. Plasma glucose levels were analyzed with the Hitachi 912 system (Roche Diagnostics), serum insulin with an immunoassay excluding des-31,32 split products and intact proinsulin (AutoDELFIA, PerkinElmer), and Hba1c by ion-exchange HPLC (B99 and BBH: Biorad; IHIT: G7, Tosoh Bioscience). Serum cholesterol, HDL-cholesterol, and triglycerides were measured using enzymatic calorimetric techniques (Roche Molecular Biochemicals). Insulin resistance was estimated by the homeostasis model assessment (HOMA-IR), calculated as [(fasting glucose level x fasting insulin level)/6.945]/ 22.5, where insulin levels were expressed as pmol/l and glucose levels as mmol/l [46]. Information about diet was obtained from validated food frequency questionnaires, as described earlier [39].

Visceral- and subcutaneous adiposity was assessed with ultrasonography according to a validated protocol, and defined as the depth in centimeters from the peritoneum to the lumbar spine, and from the skin to the linea alba, respectively. Coefficients for inter- and intra-observer variation were in the range 1.9–5.6% [47]. Fat percentage and lean mass were calculated for IHIT participants based on measures of bioimpedance from a Tanita TBF-300MA (Tanita Corporation, Tokyo, Japan).

### Genotyping

#### Stage 1 study population

The Greenlandic samples were genotyped on the Metabochip (Illumina), which contains 196,725 SNPs linked to metabolic, cardiovascular, or anthropometric traits [48]. Genotyping was performed using the HiScan system (Illumina), and genotypes were called jointly for all cohorts using the GenCall module of the GenomeStudio software (Illumina) using default cluster data. The dataset went through a two-step quality control. In step one, duplicate samples and individuals missing >2% genotypes or with gender discrepancy were removed. In step two, we removed SNPs with a minor allele frequency <1%, with >100 missing genotypes, with a large deviation from Hardy Weinberg equilibrium (p<1.0x10^-10^), as well as SNPs which were polymorphic in the IHIT cohort but not in the B99 and BBH cohorts, and SNPs associated with sex (p<1.0x10^-5^). In total, 4,674 individuals (2,791 from IHIT, 1,336 from B99, and 547 from BBH) and 115,182 SNPs passed the quality control.

#### Stage 2 study population

For the Yup’ik cohort, detailed descriptions of genotyping procedures, pedigree analyses, and data cleaning to obtain ancestry information have previously been published [49]. The rs4936356 variant was genotyped with the KASPar Genotyping assay (LGC Genomics, Hoddesdon, UK), and 1,058 individuals were available for analysis. The independent Greenlandic stage-2 cohort was genotyped on the HumanOmniExpressExome chip (8v1-2_A, Illumina) and a two-step quality control of samples and variants were carried out as described previously [45], leaving 1529 individuals for analysis.

### Imputation

To fine map the locus identified based on Metabochip data, we imputed the region. The imputation was based on Omni5Marray (Illumina) genotype data from 20 Greenlandic trios. This data was phased using ShapeIt [50], and the 40 Greenlandic parents combined with Omni 5M array data for 41 Europeans and 40 Han Chinese from the 1000 genomes project were applied as reference panel. The imputation was run with IMPUTE2 [51], where a recombination map for the reference SNPs was inferred with linear interpolation using the hg19 genomic map from IMPUTE2 as a template, and an effective population size of 1500. Imputed genotypes with an info score above 0.4 were analyzed as dosages using GEMMA, for details see below.

### Statistical analysis

#### Stage 1 association testing

To account for relatedness and admixture, we applied a linear mixed model, implemented in the software GEMMA [52], for association testing. For each phenotype, the tests were applied to data from all individuals across the three cohorts with information about that specific phenotype, and the relatedness matrix required as input to GEMMA was estimated from genotypic data from these individuals only. For all tests, we assumed an additive effect and included sex, age, and cohort as covariates. Prior to performing association tests, quantitative traits were quantile transformed to a standard normal distribution within each sex. Individuals with previously diagnosed diabetes were excluded from analyses of quantitative traits, and individuals taking lipid-lowering drugs were excluded from analyses of fasting serum lipids. For BMI, we also performed a recessive association analysis using the same criteria as described for the additive analysis.

The Greenlandic population is an admixture of Inuit and Europeans, and we applied the asaMap method [19] to estimate the effect size of the BMI-associated variant in each ancestry component of the study population, and to compare the contribution from each ancestry component to the association. With asaMap, we ran a linear regression applying an additive model adjusted for age, sex, cohort, and the first 10 principal components to account for the relatedness and population structure.

#### Stage 2 association testing–replication analyses and meta-analysis

The Yup’ik cohort was also analyzed with the GEMMA software [52]. For this data, the genetic similarity matrix required for the association analysis was calculated using the genotype data from the linkage panel merged with the additional genotypes of the SNP genotyped for this study. The admixture with Caucasian populations in this cohort was negligible [53], making admixture estimation unnecessary. Allele frequencies for rs4936356 were estimated using the MENDEL program [54].

Association testing in the independent Greenlandic stage-2 cohort was also done using the linear mixed effects model implemented in the GEMMA software [52] to account for relatedness and admixture. The relatedness matrix required as input to GEMMA was estimated from genotype data from all autosomal variants with minor allele frequency >5% and <1% missing genotypes. Prior to performing the association test, BMI was quantile transformed to a standard normal distribution within each sex. The association test was performed assuming an additive genetic model, with sex and age as covariates.

We performed a meta-analysis of the results from the stage 1 population and the two stage 2 replication cohorts based on the estimated effect sizes and their standard errors in METAL [55]. Heterogeneity between cohorts was assessed with Cochran’s Q test statistics [56].

### Estimation of ancestral allele frequencies

We estimated the allele frequency of rs4936356 separately for the Inuit and European ancestry components of the admixed Greenlandic population applying a two-step approach. In step 1, ancestry proportions for the Greenlandic individuals from the stage 1 study population, as well as for 50 Danish individuals, were estimated using ADMIXTURE v1.3.0 [57], assuming two ancestral populations—Inuit and Europeans. In step 2, ancestral allele frequencies with confidence intervals for each SNP separately using bootstrap with replacement were estimated. We used 1000 bootstrap samples of individuals and performed maximum likelihood estimation of the allele frequencies, using the likelihood function from ADMIXTURE with the ancestry proportions fixed to the estimates obtained in step 1. The confidence intervals were based on the quantiles of these bootstrap estimates.

### Assessment of possible functional effects

#### RNA expression analyses

Whole transcriptome RNA was extracted in 2.5 ml peripheral blood from 499 Greenlanders from the stage 1 study population. The extraction was performed with the PAXgene Blood miRNA kit according to the manufacturer’s protocol, and subjected to on-column DNase I treatment with RNase-free DNase (Qiagen, Hilden, Germany). The RNA quality and purity were assessed using an Agilent 2100 Bioanalyzer (Agilent RNA 6000 Nano Kit) and NanoDrop, respectively.

TruSeq RNA Sample Prep Kit v2 (Illumina) was used to prepare the RNA sequencing library. Isolation of mRNA was carried out with oligo(dT) beads on 200 ng of total RNA, and fragmentation with Elute, Prime, Fragment Mix. First-Strand Mix and SuperScript II (Invitrogen) reverse transcription master mix was applied for generation of first-strand cDNA, and the second strand was synthesized by adding Second-Strand Master Mix. End-repairing and purification of the fragmented cDNA were performed with AMPure XP Beads (Agencourt), and A-Tailing Mix was added, and reactions were incubated. For adaptor ligation, Adenylate 3′ Ends DNA, RNA Index Adaptor and Ligation Mix were mixed and reactions were incubated. End-repaired DNA was purified with AMPure XP Beads (Agencourt). PCR amplification with PCR Primer Cocktail and PCR Master Mix were performed to enrich the cDNA fragments, and PCR products were purified with AMPure XP Beads (Agencourt). Agilent 2100 Bioanalyzer instrument (Agilent DNA 1000 Reagents) and by real-time qPCR (TaqMan Probe) were used to measure the average molecule length. The qualified libraries were amplified on a cBot to generate the cluster on the flow cell (TruSeq PE Cluster Kit V3–cBot–HS, Illumina).

Amplified libraries were sequenced using the BGI500 sequencing technology at BGI (100bp paired-end sequencing). We assessed the quality of the sequencing reads using FastQC [58], and inspected the aggregated results using multiQC [59]. Sequencing adapters and low-quality reads were removed using trimmomatic [60]. After trimming, we reassessed the quality of the sequencing data using FastQC. A total of 17–49 (median: 21) million read pairs passed the quality filters and was used for expression quantification. Transcript level quantification was obtained by pseudo-mapping to Ensemble v.94 (GRCh38) annotation using kallisto [61]. Transcript level expression (TPM) was aggregated to gene level expression using tximport [62]. Lastly, gene level expression was quantile normalized. We tested for association between gene expression levels for a set of genes neighboring the variant (rs4936356) by applying a linear mixed model, as implemented in GEMMA [52], where we accounted for genetic relatedness and admixture. Gender and age were included as covariates in the analyses.

#### In-silico analyses

The RegulomeDB [20] and HaploReg V4.1 [21] databases were queried to assess co-localization with regulatory elements, such as transcription factor binding sites, promoter regions, and regions of DNase hypersensitivity. Moreover, RNA expression data from 48 tissues (with >70 samples, range: 80–399) were queried through the GTEx Portal (https://www.gtexportal.org/; accessed 14-06-2019) to assess possible effects of the genetic variant on the expression of nearby genes.

## Data availability

The Greenlandic Metabochip-genotype data and the RNA sequencing data are deposited in the European Genome-phenome Archive (https://www.ebi.ac.uk/ega/home) under the accessions EGAS00001002641 and EGAS00001004127, respectively.

## Supporting information

S1 Fig Manhattan plot and QQ plot for stage 1 additive association of Metabochip variants with BMI. The dashed line in the Manhattan plot indicates the genome-wide significance threshold of p = 5x10^-8^. P-values were calculated based on data transformed to a standard normal distribution.
(PDF)

S2 Fig Manhattan plot and QQ plot for stage 1 recessive association of Metabochip variants with BMI. P-values were calculated based on data transformed to a standard normal distribution.
(PDF)

S3 Fig Regional BMI-association results conditional on rs4936356. The association analysis was based on imputed data, and the dark red dot indicates the lead SNP in the region (rs4936356). The rest of the SNPs are colored according to the extent of correlation (*r*^2^) with the lead SNP.
(PDF)
