# Supplementary figures and images for: The derived allele of a novel intergenic variant at chromosome 11 associates with lower body mass index and a favorable metabolic phenotype in Greenlanders
Source: PLoS Genet. 2020 Jan 24;16(1):e1008544. doi: 10.1371/journal.pgen.1008544 (PMC7001991; doi:10.1371/journal.pgen.1008544)

S1 Fig.

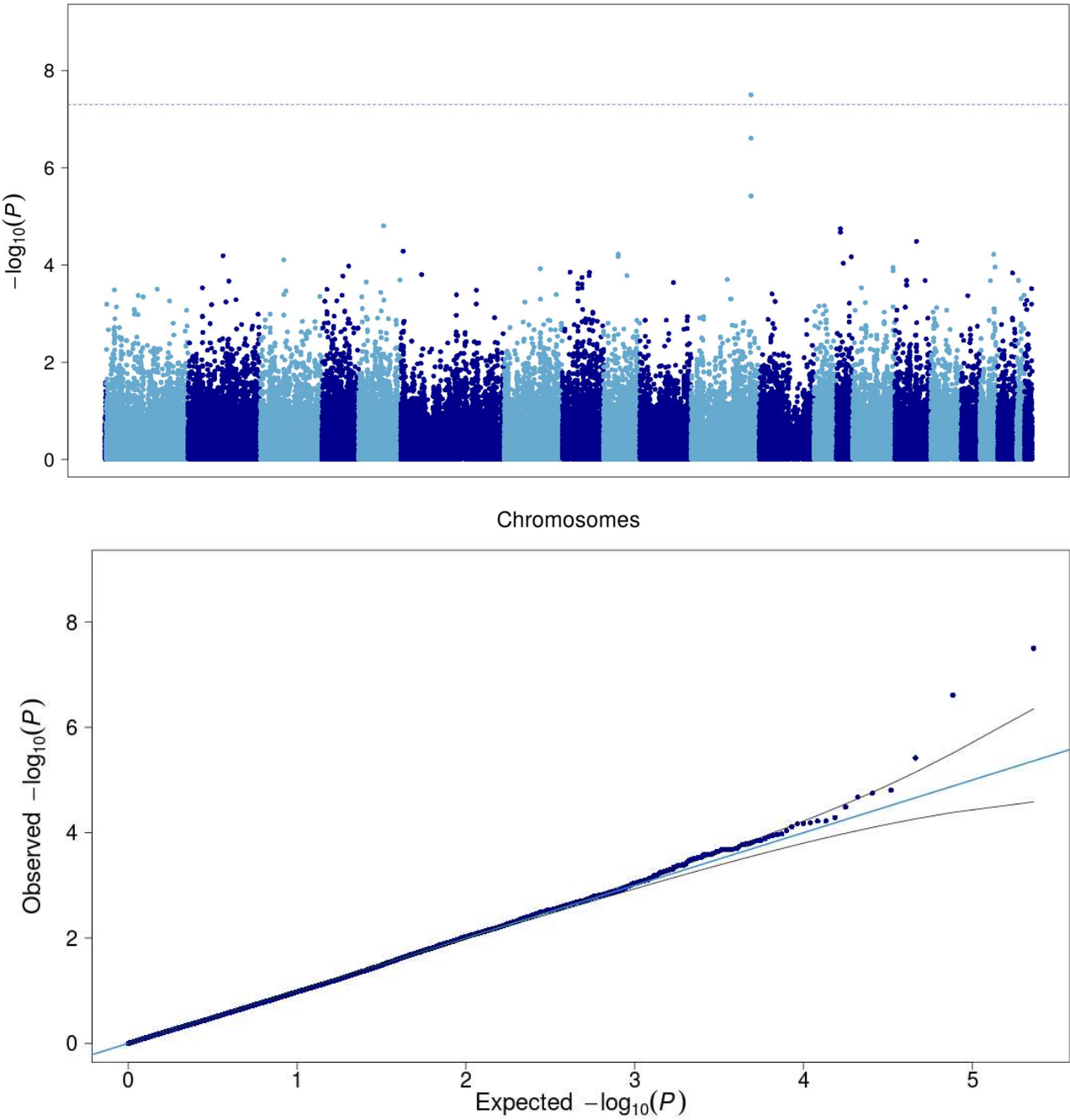

Supplement: S1 Fig — The dashed line in the Manhattan plot indicates the genome-wide significance threshold of p = 5x10^-8^. P-values were calculated based on data transformed to a standard normal distribution. (PDF) [file pgen.1008544.s001.pdf]

S2 Fig.

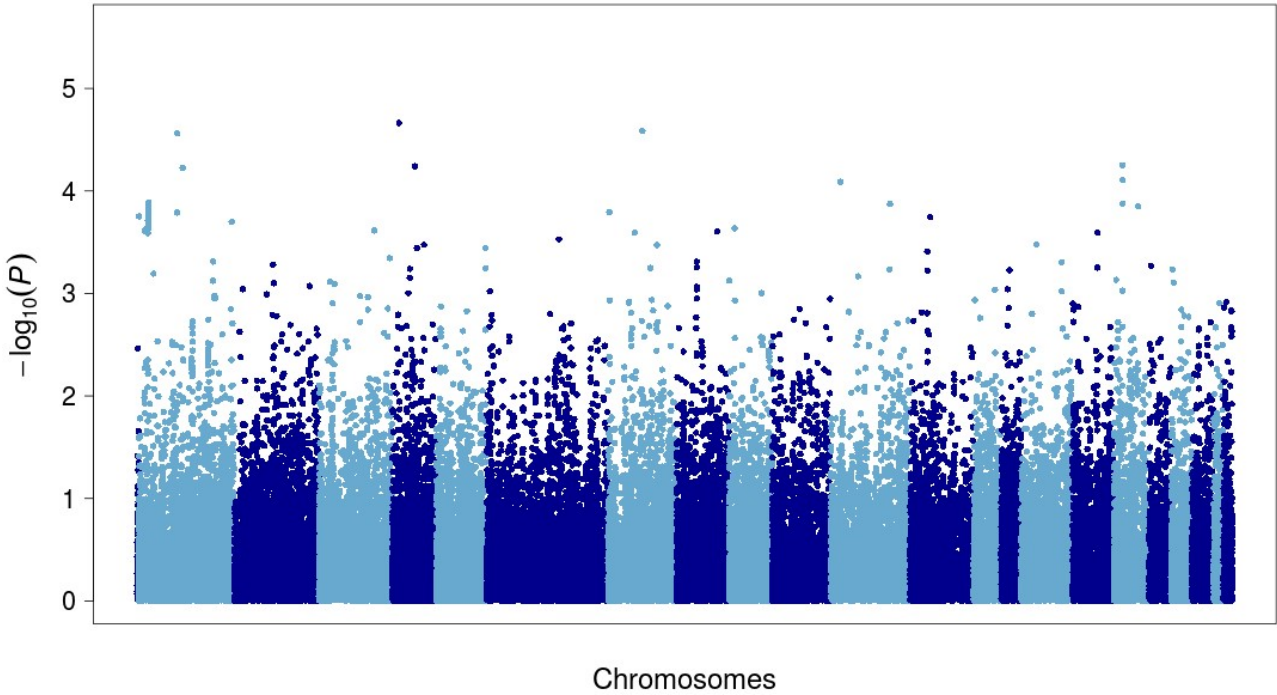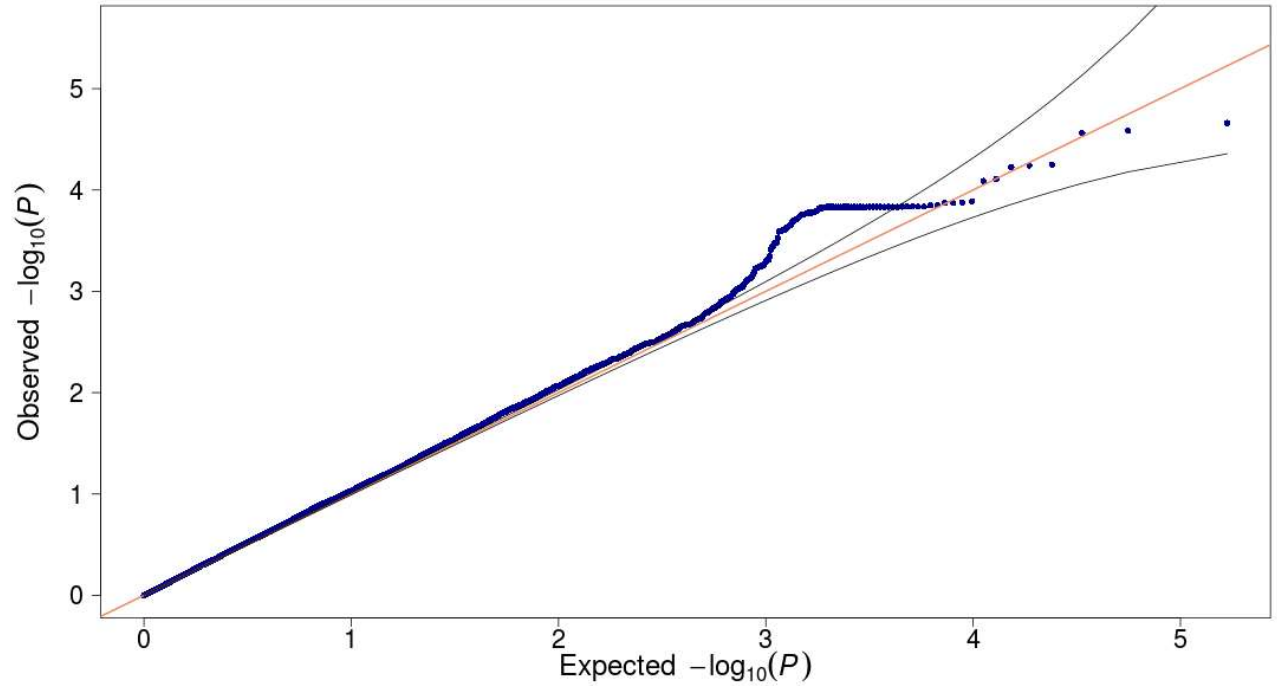

Supplement: S2 Fig — P-values were calculated based on data transformed to a standard normal distribution. (PDF) [file pgen.1008544.s002.pdf]

S3 Fig.

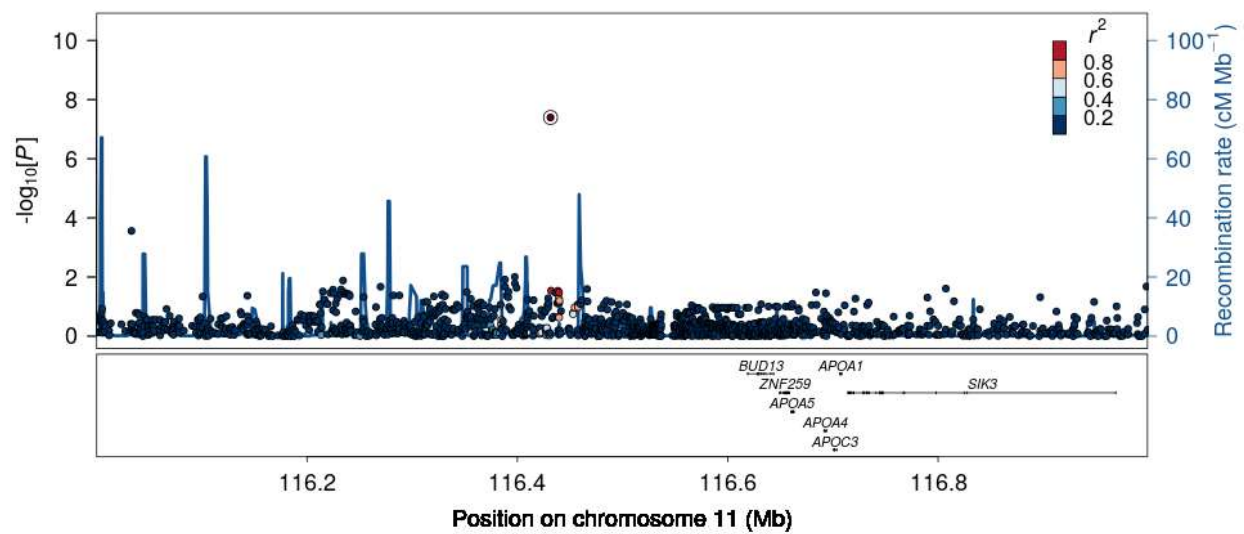

Supplement: S3 Fig — The association analysis was based on imputed data, and the dark red dot indicates the lead SNP in the region (rs4936356). The rest of the SNPs are colored according to the extent of correlation (r2) with the lead SNP. (PDF) [file pgen.1008544.s003.pdf]
